# Genotyping of *Trypanosoma brucei evansi* in Egyptian camels: detection of a different non-RoTat 1.2 *Trypanosoma brucei evansi* in Egyptian camels

**DOI:** 10.1007/s11250-023-03673-6

**Published:** 2023-07-28

**Authors:** Tahani Salama Behour, Eman Mohamed Abd EL Fattah

**Affiliations:** Biotechnology Research Unit, Animal Reproduction Research Institute, Agricultural Research Center, 5 Bohooth El-Hadaek Street, Al Haram, P.O. Box 12556, Giza, Egypt

**Keywords:** *T. B. evansi*, Egyptian camels, TBR PCR, ITS-1 PCR, JN 2118Hu PCR, VSG RoTat 1.2 PCR, Non-RoTat1.2, EVAB PCR

## Abstract

*Trypanosoma brucei evansi* (*T. b. evansi*) is an enzootic organism found in Egyptian camels, which genetically classified into types A and B. To detect the parasite genotype circulating in Egyptian camels, we collected 94 blood samples from three distant districts and subjected them to different PCR assays; *T. brucei* repeat (TBR), internal transcribed spacer-1 (ITS-1), and variable surface glycoproteins (VSG) (RoTat 1. 2, JN 2118Hu) and EVAB PCRs. The highest prevalence was obtained with TBR (80/91; 87.9%), followed by ITS-1 (52/91; 57.1%), VSG JN 2118Hu (42/91; 46.2%), and VSG RoTat 1. 2 (34/91; 37.4%). We reported a different non-RoTat 1. 2 *T. b. evansi* for the first time in Egyptian camels. Results showed that 47 (58.7%) out of 80 samples were classified as *T. b. evansi*. Of these, 14 (29.8%) were RoTat 1. 2 type, 13 (27.6%) were non-RoTat 1. 2 type, and 20 (42.6%) samples were from mixed infection with both types. All samples were tested negative with EVAB PCR. RoTat 1. 2 *T. b. evansi* was the most prevalent in Giza and El Nubariyah, whereas, in Aswan, the only type detected was non-RoTat 1. 2 *T. b. evansi.* The nucleotide sequences of the VSG RoTat 1.2 and JN 2118Hu PCR products were submitted to DNA Data Bank of Japan (DDBJ) and GenBank under the accession numbers LC738852, and (OP800400-OP800403). Further research is required to increase the sample size and verify the new sequences to corroborate the prevalence of a new variant of non-RoTat 1.2 *T. b. evansi* in Egypt.

## Introduction

*Trypanosoma brucei evansi*, belonging to the family *Trypanosomatidae*, is a unicellular protozoan parasite distributed in the blood of wild and domestic animals but rarely in humans (Joshi et al. 2005; Desquesnes et al. 2013). Previously, *T. evansi* and *T. equiperdum* were classified as separate species but recent evolutionary studies proposed that *T. evansi* and *T. equiperdum* originate from genetically diverse *T. brucei* strains and hence, are *T. brucei* subspecies (Lai et al. 2008; Carnes et al. 2015). It causes a wasting disease called surra that imposes significant economic losses due to reduced fertility, productivity, and mortality of untreated animals (Desquesnes et al. 2013).

Trypanosomes can be distinguished by the presence of kinetoplasts containing DNA (kDNA) that matches their mitochondrial DNA. The *T. brucei* kDNA is characterized by 20 to 50 maxicircles (approximately 23 kb) together with thousands of extremely varied minicircles (approximately 1 kb). Due to a partial loss of kDNA, *T. b. equiperdum* and *T. b. evansi* are termed dyskinetoplastic. *T. b. evansi* exhibits homogeneous minicircles. Although *T. b. equiperdum* retains its maxicircles, occasionally with significant deletions, it has lost the diversity of its minicircles (Claes et al. 2005; Lai et al. 2008; Schnaufer 2010).

According to the restriction digestion pattern of the minicircles, *T. b. evansi* is distinguished into types A and B (Njiru et al. 2006), with the former being more prevalent. Distributed in Africa, Asia, and South America, it is defined by the gene for the variable surface glycoprotein (VSG) RoTat 1.2. Its antibodies can be detected in *T. b. evansi* type A-infected animals as VSG is expressed during the early infection stages (Verloo et al. 2001). *T. b. evansi* type B is relatively less frequent and was initially detected in Africa in camels from Kenya (Borst et al. 1987; Ngaira et al. 2005). Lately, it has been reported in Ethiopia (Birhanu et al. 2016) then, more recent work by Oldrieve et al. (2021) using genomic data from various Trypanozoon strains, reclassifies the IVM-t1 strain from Mongolia as the first Asian *T. brucei evansi* type B strain.

As the RoTat 1.2 gene sequence is missing in *T. b. evansi* type B, serological and molecular assays targeting RoTat 1.2 VSG, including the CATT/ RoTat 1.2 and RoTat 1.2 PCR cannot detect type B infections (Claes et al. 2004). So far, three molecular assays have been established for identifying *T. b. evansi* type B: (1) EVAB PCR: based on a minicircle DNA sequence specific to type B, (2) a PCR, and (3) a LAMP, both based on VSG JN 2118Hu type B-specific sequence (Ngaira et al. 2005; Njiru et al. 2010). In Egypt, *T. b. evansi* is endemic in camels and horses, while water buffaloes are considered reservoirs (Elhaig and Sallam 2018). Previous molecular studies reported that RoTat1.2 VSG PCR could not detect *T. b. evansi* in some infected animals from different species (Elhaig and Sallam 2018; Behour et al. 2019). Therefore, in this study, our objective was to detect and identify the trypanosome type, specifically for *T. b. evansi,* found in Egyptian camels, using type-specific molecular tools.

## Materials and methods

### Ethical statement

The animal manipulation and sample collection methods used in this study were approved by The Ethical Research Committee of the Animal Reproduction Research Institute (ARRI), Agricultural Research Center (Code No.1 14 1 4 2 9).

### Samples collection

**Fig. 1:**
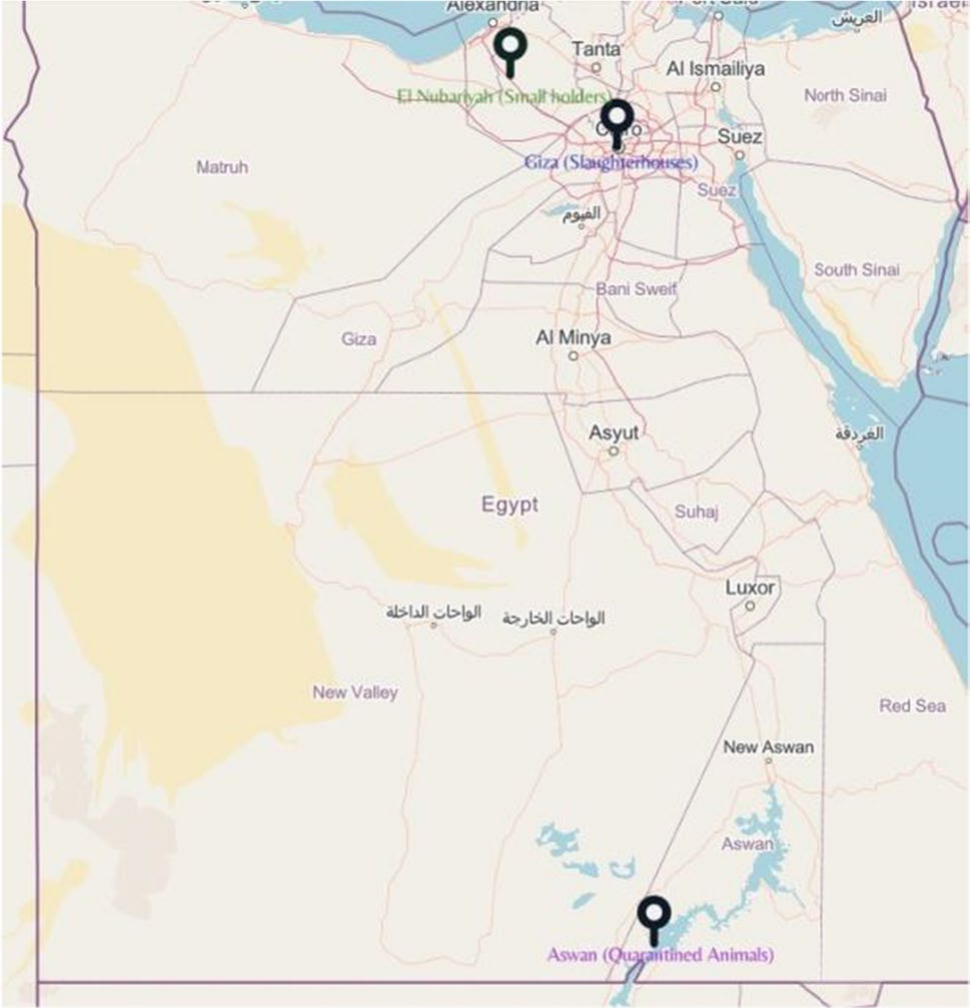
Map of Egypt shows the locations from which camels blood samples were collected. Samples were collected from small holders in El Nubariyah (20 samples), slaughterhouses in Giza (43), quarantined animals in Aswan (26 samples). The generated map was done using ARcGris online from Esri, Map data © OpenStreetMap contributors, Microsoft, Facebook, and its affilates, Esri community Map contributors, map layer by Esri

We collected 94 blood samples from adult apparent healthy camels in the districts of Aswan (26 samples from quarantined animals), Giza (43 samples from slaughterhouses), and El Nubariyah (20 samples from small holders) as shown in Fig. 1. The blood samples (3 ml/camel) were collected in EDTA by jugular vein puncture. Then, the samples were transferred on ice to the laboratory for molecular analysis.

### DNA extraction

Genomic DNA was extracted from the whole blood samples using GeneDirex genomic DNA Isolation Kit (GeneDirex, Inc., Taiwan) following the manufacturer’s instructions. DNA quantity and purity were measured using a spectrophotometer (BioPhotometer plus, Eppendorf, Hamburg, Germany) and stored at − 20 °C until further use. The positive control DNA of *T. b. evansi* RoTat1.2 was previously isolated from naturally infected camels and was stored at − 20 °C (Behour et al. 2019). Also DNA of DEAE purified* T. b. evansi* type B was kindly provided by Institute of Tropical Medicine, Antwerp, Belgium.

### PCR assays

The DNA isolated from each sample was subjected to different PCR assays for detecting and typing the trypanosomes. Table 1 indicates each PCR assay’s target, primers sequence, amplicon size, annealing temperature, and reference. *T. brucei* repeat (TBR) PCR assay is the gold standard for detecting Trypanozoon in addition to Internal transcribed spacer-1 (ITS-1) PCR. VSG RoTat 1.2 and JN 2118Hu PCRs ( for RoTat 1.2 and non-RoTat 1.2 T*. b. evansi*, respectively) were applied to detect *T. b. evansi* VSG genes, and EVAB PCR targeting class B minicilcles for *T. b. evansi* type B. Moreover, all the VSG JN 2118Hu-positive samples were tested with maxicircle gene NADH-dehydrogenase subunit (ND5) PCR to exclude other *T. brucei* spp. The success of extraction method was tested with DRB-Exon2 PCR for camel DNA on the TBR-negative samples. The same, a single copy gene namely a phospholipase C (GPI-PLC) PCR was performed for samples that are RoTat1.2 negative to demonstrate that enough parasite material was available for amplification. For each assay, the amplification mixture (25 µl) consisted of Dream Taq^TM^ Green PCR Master Mix (2x) (Thermo Scientific, Lithuania), 25 pmol of each assay-specific primer, and 100 ng of DNA template. Positive and negative controls were also included in each PCR assay. Amplification was performed using the SimpliAmp thermocycler (Applied Biosystems, Thermo Fisher Scientific, Singapore) using the following conditions: initial denaturation for 5 min at 95 °C, followed by 35 cycles of denaturation at 94 °C for 45 s, the specified annealing for 45 s, extension at 72 °C for 60 s, and finally, an extension cycle at 72 °C for 10 min. Each amplicon (7 µl) was detected using electrophoresis on 1.5% agarose gel and then visualized under ultraviolet light after staining with ethidium bromide.

**Table 1:** Details of the PCR assays performed for each sample

Target gene (organism)	Primer sequence	Amplicon size	Annealing temperature	Reference
DRB-Exon2 (camel)	DRB-Exon2F5′-AGCAGTGGGGGTCCTAGTG-3′ DRB-Exon2R5′ACCCACCCGGACTCAGTATC-3′	457 bp	55 °C	Plasil et al. 2016
GPI-PLC gene (Trypanozoon)	657 F5′-CGCTTTGTTGAGGAGCTGCAAGCA-3′ 658R5′-TGCCACCGCAAAGTCGTTATT TCG-3′	324 bp	60 °C	Picozzi et al. 2008
TBR (Trypanosomes)	TBR F 5′-GAA TAT TAA ACA ATG CGC AG-3′ TBR R 5′-CCA TTT ATT AGC TTT GTT GC-3′	164 bp	52 °C	Masiga et al. 1992
ITS-1 internal (Trypanozoon)	F-5′ ATAAATTGCACAGTATGCAACCAAA-3′ R-5′CATCCCTCATCTCCCATGTCA-3′	90 bp	60 °C	Taylor et al. 2008
VSG RoTat 1.2 (*T. b. evansi *type A)	TeRoTat920 F 5′-CTG AAG AGG TTG GAA ATG GAG AAG-3′ TeRoTat1070 R 5′-GTT TCG GTG GTT CTG TTG TTG TTA-3′	151 bp	60 °C	Konnai et al. 2009
Minicircle class B (*T. b. evansi* type B	EVAB1 5'-CACAGTCCGAGAGATAGAG-3' EVAB2 5'-CTGTACTCTACATCTACCTC-3'	436 bp	60 °C	Njiru et al. 2006
VSG JN 2118Hu (non-RoTat 1.2 *T. b. evansi*)	F-5′TTCTACCAACTGACGGAGCG-3′ R-5′TAGCTCCGGATGCATCGGT-3′	273 bp	55 °C	Ngaira et al. 2005
Maxicircle ND5 (*T. brucei*)	F-5′ TGGGTTTATATCAGGTTCATTTATG-3′ R-5′ CCCTAATAATCTCATCCGCAGTACG-3′	400 bp	54 °C	Dean et al. 2013

### Sequencing of PCR products

The PCR amplicons of *T. b. evansi* RoTat 1.2 (2 samples) and non-Rotat 1.2 (4 samples) were purified using Gene JET Gel Extraction Kit (Thermo Scientific K0691, Germany) according to the manufacturer’s instructions. We performed forward and reverse sequencing in an automated DNA sequencer (ABI 3730XL, Applied Biosystems, USA). The results were compared with available NCBI GenBank sequences and submitted to DNA Data Bank of Japan (DDJB) and GenBank. The sequences were aligned using Clustal W with the BioEdit program v7.2.5 (Hall 1999). The phylogenetic tree was constructed using the Maximum Likelihood method and Jukes–Cantor model (Jukes and Cantor 1969) with 1000 bootstrap replicates conducted using Mega 11 (Tamura et al. 2021).

According to Ngaira et al. (2005), the restriction sites of *Hae*III enzyme in the sequenced non-RoTat1.2 PCR fragment were determined using the NEB cutter v3.0 program: https://nc3.neb.com/NEBcutter.

## Results

**Fig. 2:**
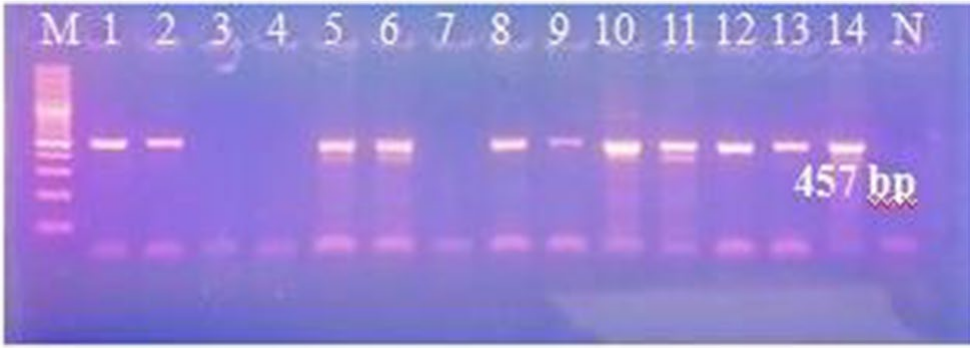
Agarose gel image of DRB-Exon2 PCR products amplified from camel DNA. It shows successful amplification of 457-bp DNA fragment compared with 100-bp DNA ladder in 11 samples out of 14 TBR-negative samples

**Fig. 3:**
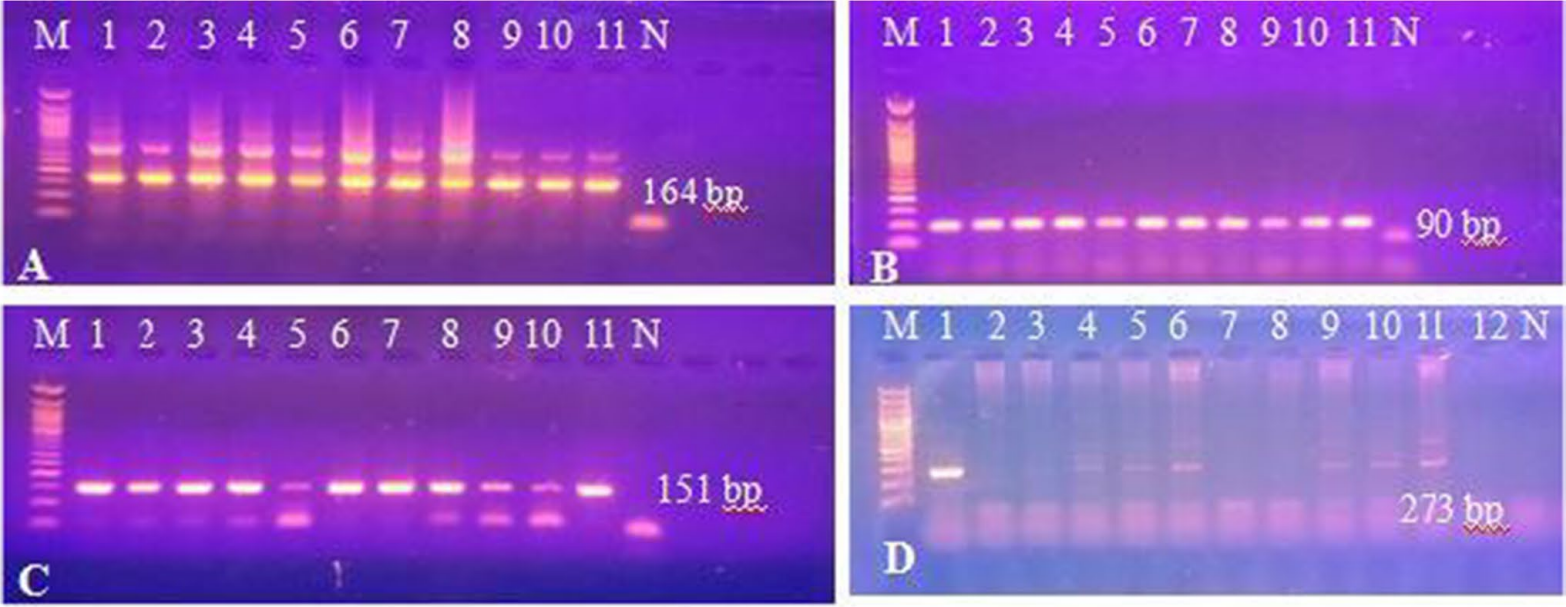
Agarose gel image of the PCR products showing detection of *T. b. evansi* in the camels’ blood. **A** Amplified products of TBR PCR (164 bp); M, 50-bp ladder; lanes 1–11, positive samples; N, negative control. **B** Amplified products of ITS-1PCR (90 bp); M, 50-bp ladder; lanes 1–11, positive samples; N, negative control. **C** Amplified products of RoTat 1.2. PCR (151 bp); M, 50-bp ladder; lanes 1–11, positive samples; N, negative control, and **D** Amplified products of VSG JN 2118Hu PCR(273 bp); M, 100-bp ladder; lane 1– *T. b. evansi* type B-positive control; lanes 2–11, some tested samples; 12; RoTat1.2-positive sample; N, negative control

**Fig. 4:**
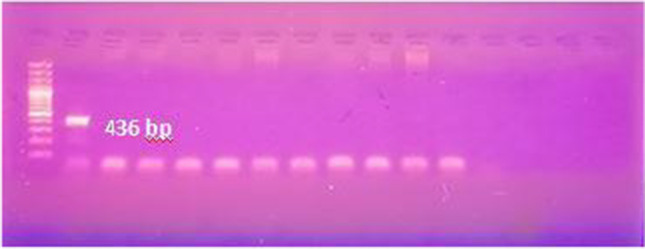
Agarose gel image of the EVAB PCR products for detection of *T. b. evansi* type B. It shows negative results of tested samples compared with *T. b. evansi* type B-positive control (436 bp) and 100-bp DNA ladder

**Fig. 5:**
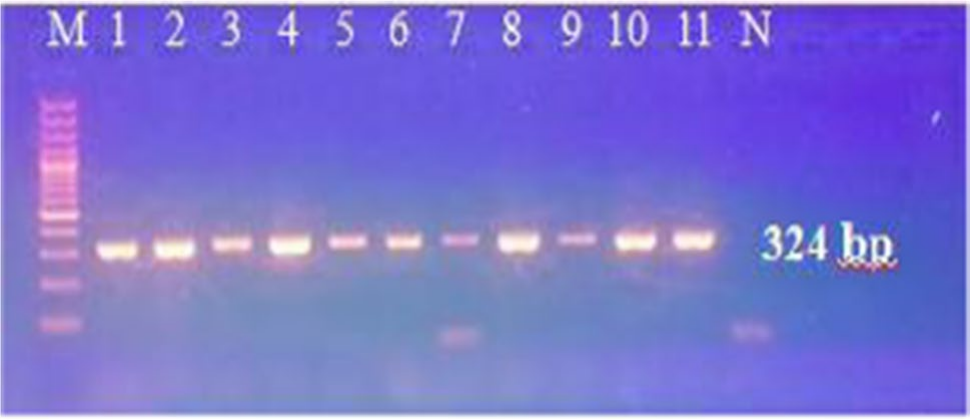
Agarose gel image of GPI-PLC PCR products of some RoTat1.2 and JN 2118Hu PCR-positive samples for detection of a single copy control gene. It shows amplification of 324-bp DNA fragment of *T. b. evansi* compared with a 100-bp DNA ladder

**Fig. 6:**
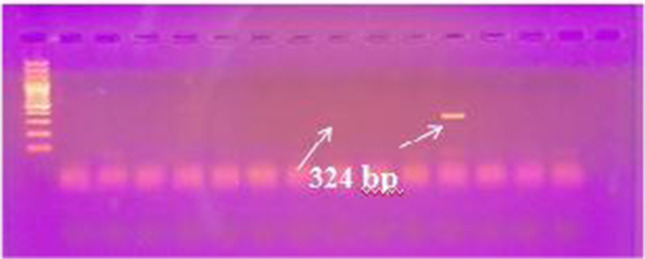
Agarose gel image of GPI-PLC PCR products of RoTat1.2 and JN 2118Hu PCR negative samples for detection of a single copy control gene. It shows amplification of 324-bp DNA fragment in only two samples (one of them is very faint) compared with a 100-bp DNA ladder

We performed different PCR assays to detect *T. b. evansi* in the sample DNA. Using TBR PCR, a specific 164-bp band was obtained in 80 out of 94 samples. Of the 14 negative samples, 3 were tested negative for camel DNA with DRB-Exon2 PCR (Fig. 2) and therefore were excluded from results calculations. Of the 91 successfully extracted samples, 52 were positive with ITS-1PCR, 34 with VSG RoTat 1.2 PCR, and 42 samples with VSG JN 2118Hu PCR (Fig. 3A–D), while none tested positive with ND5 PCR or EVAB PCR (Fig. 4). Two out of 25 RoTat 1.2 PCR negative samples other than JN 2118Hu PCR-positive samples, gave positive amplification with GPI-PLC PCR (Figs. 5 and 6). Table 2 indicates the overall prevalence of *T. b. evansi* for each PCR assay. The highest prevalence was with TBR (87.9%), while the lowest was with RoTat 1.2.PCR (37.4%).

**Table 2:** The molecular prevalence of *T. b. evansi* detected using PCR assays

PCR assay	No. of positive samples	No. of negative samples	Total, successfully DNA extracted, tested samples	Prevalence (%)
TBR	80	14	91	87.9
ITS-1	52	42	91	57.1
VSG RoTat 1.2	34	60	91	37.4
VSG JN 2118Hu	42	52	91	46.2
EVAB	0	91	91	0

The detected trypanosomes were typed by comparing the results of the four PCR assays (Table 3). We obtained positive amplification with 47 (58.7%) of the 80 samples classified as *T. b. evansi* positive with TBR, ITS-1, and RoTat or JN 2118Hu PCR. Of these 47 samples, 14 were identified as RoTat 1.2 *T. b. evansi*, and 13 samples were typed as non-RoTat1.2 *T. b. evansi*, which displayed positive amplification with RoTat 1.2 and JN 2118Hu PCR, respectively, whereas 20 samples showed mixed infection with both types. The 33 samples that are positive on both TBR and ITS-1 were classified as Trypanozoon. Regarding the prevalence of *T. b. evansi* in each district (Table 4), its prevalence was highest in Giza (27.5%), with RoTat1.2 *T. b. evansi* being predominant. Whereas the lowest prevalence (11%) was reported in Aswan, where the only type recorded was non-RoTat 1.2 *T. b. evansi*.

**Table 3:** Typing of *T. b. evansi–*positive samples according to results of the four PCR assays

No. of samples	TBR	ITS-1	VSG RoTat 1.2	VSG JN 2118Hu	Typing	Total	Percentage (%)
19	** + **	** − **	** − **	** − **	**Trypanozoon**	**33/80 (41.3%)**	**57.6**
5	** + **	** + **	** − **	** − **	**Trypanozoon**		**15.1**
9	** + **	** − **	** − **	** + **	**Trypanozoon**		**27.3**
14	** + **	** + **	** + **	** − **	***RoTat 1.2 T. b. evansi***	**47/80 (58.7%)**	**29.8**
13	** + **	** + **	** − **	** + **	***Non-RoTat 1.2 T. b. evansi***		**27.6**
20	** + **	** + **	** + **	** + **	***Mixed T. b. evansi***		**42.6**

**Table 4:** Total positive samples and typing of *T. b. evansi* from each district

District	No. of tested samples	*T. b. evansi* positive (%)	RoTat1.2* T. b. evansi*	non-RoTat1.2* T. b. evansi*	*T. b. evansi* *(mixed)*
El Nubariyah	20	12 (13.2%)	3	1	8
Giza	43	25 (27.5%)	11	2	12
Aswan	28	10 (11%)	–	10	–
Total	91	47 (52%)	14	13	20

**Fig. 7:**
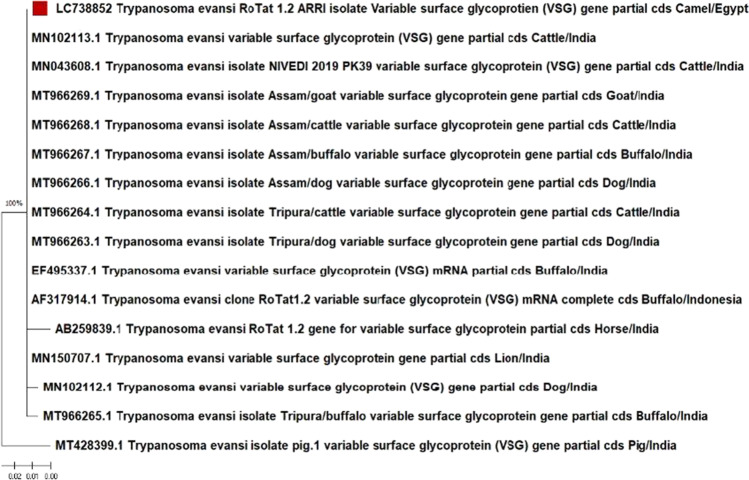
The original phylogenetic tree constructed using 14 partial cd sequences of *T. b. evansi* RoTat 1.2 and one mRNA complete cd sequence using the Maximum Likelihood method and Jukes–Cantor model (Jukes and Cantor 1969) with 1000 bootstrap replicates conducted in Mega 11 (Tamura et al. 2021). The *T. b. evansi* RoTat 1.2 type sequences with accession numbers, isolate names, host names, and country where it was isolated, are listed. LC738852 is the accession number of the sequence for our isolate *Trypanosoma evansi* ARRI VSG gene for variable surface glycoprotein, partial cds indicated by the red square

We analyzed the sequencing results of VSGRoTat 1.2 PCR products for *T. b. evansi* and submitted it to the DDJB as *Trypanosoma evansi* ARRI VSG gene for variable surface glycoprotein, partial cds, under the accession number LC738852. Using the nucleotide basic local alignment search tool (nBLAST), we found that the RoTat sequence had 96.03% to 100% sequence identity with all the *T. b. evansi* VSGRoTat 1.2 sequences from GenBank with high specificity for *Trypanosoma brucei evansi* RoTat 1.2. The 100% identity was found with *T. evansi* clone RoTat 1.2VSG (AF317914), which was originally isolated from a *T. b. evansi* strain from water buffalo in Indonesia, and *T. evansi* VSG partial cds isolated from cattle (accession no.: MT966264; MT466268; MN043608; MN1012113), buffalo (EF495337; MT966267), goat (MT966269), dog (MT966263; MT966266) in India. We also found 96.03%, 98.68%, 99.34%, and 99.34% identity with *T. b. evansi* VSG cds isolated from pigs (accession no: MT428399), horses (AB259839), buffaloes (MT966265), and dogs (MN102112), respectively, from India. This sequence was not previously recorded from camel origin in the GenBank database. Our isolate was significantly related to the Indian and Indonesian isolates according to the phylogenetic tree (Fig. 7), which showed a close relationship among *T. b. evansi* targeting RoTat 1.2 VSG.

**Fig. 8:**
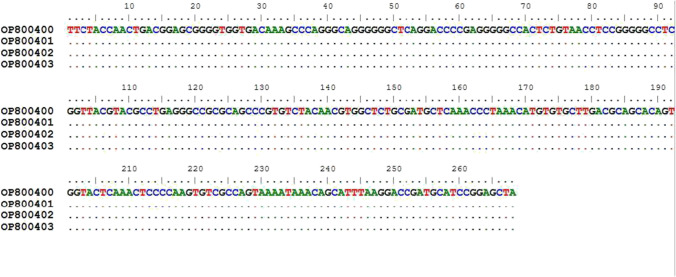
Multiple sequence alignment of the sequences of the four sample isolates (OP800400, OP800401, OP800402, OP800403) amplified by VSG JN 2118Hu PCR assay. The sequences were aligned using Clustal W conducted in BioEdit program v7.2.5 (Hall 1999), which indicated that the isolates were identical and sequences were repetitive

The sequencing results of the VSG JN 2118Hu PCR products for non-RoTat 1.2 *T. b. evansi* were analyzed and submitted to GenBank as *Trypanosoma evansi* isolate Egy_ARRI1, 2, 3, 4 sequences under the accession numbers: OP800400; OP800401; OP800402; OP800402; OP800403. The nBLAST results for the VSG JN 2118Hu PCR sequences indicated a lack of similarity with all sequences obtained from GenBank (except primers sequence region). However, as the four sequence isolates were identical (Fig. 8), they were submitted as unverified sequences. The NEBcutter computer program exposed three restriction sites of *Hae* III enzyme in the sequence of non-RoTat1.2 PCR fragment. The restricted fragments were 149 bp (right end), 67 bp (left end), 31 bp, and 21 bp.

## Discussion

Recently, molecular diagnostic tests have been used instead of parasitological methods for detecting *T. b. evansi* to expedite diagnosis, lower cost, and improve our epidemiological understanding (Birhanu et al. 2015). TBR PCR was carried out using TBR primers to amplify a 164 bp highly repeated minichromosome satellite DNA sequence (Masiga et al. 1992). This test efficiently detected 80 positive samples out of 94 tested samples (87.9%). The high sensitivity of this PCR has been previously reported (Fernandez et al. 2009; Elhaig and Sallam 2018; Behour et al. 2019). The TBR primers were the first to be described and are primarily used for detecting Trypanozoon DNA. They are regarded as the “gold standard” for identifying *T. b. evansi* to avoid the issues related to the failure of the primers targeting kDNA. This can be attributed to the fact that TBR primers target highly repetitive satellite sequences (approximately 10,000–20,000), which results in a highly sensitive PCR test that is independent from the presence of parasite kDNA (Ventura et al. 2000; Fernandez et al. 2009). The ITS-1 PCR assay targets ITS-1, positioned between 18S and 5.8S ribosomal DNA sequences. Its size is species-specific, which enables the detection and identification of polyspecific infections (Desquesnes et al. 2001). The ITS-1 internal primers successfully amplified a 90-bp Trypanozoon DNA fragment (Taylor et al. 2008), which helped identify 52/91 (57.1%) positive samples. Since the primers were designed for detecting *T. b evansi* (Taylor et al. 2008) and as the genome of *T. b. evansi* contains lower copies (~ 100–200) of the ITS regions (Fernandez et al. 2009) compared with TBR, the sensitivity of this assay was lower than TBR PCR.

In the RoTat 1.2 PCR assay, a primer pair for *T. b. evansi* type A, targeting the *T. b. evansi* RoTat 1.2 VSG gene, was used to amplify a 151-bp DNA fragment (Konnai et al. 2009). Although this specific DNA region lacks homology with other known VSG genes in trypanosomes, it is highly conserved among the *T. b. evansi* strains (Claes et al. 2004). We confirmed that 34/91 (37.1%) were *T. b. evansi* RoTat 1.2-positive using RoTat 1.2 PCR. Although most strains of *T. b. evansi* type A carry RoTat 1.2 VSG gene (Urakawa et al. 2001; Ngaira et al. 2005), some strains of *T. b. evansi* type A and *T. b. evansi* type B lack it (Ngaira et al. 2004, 2005; Kamidi et al. 2017). Furthermore, *T. b. evansi* strains can completely lose their kinetoplast, which would produce a false-negative result in a diagnostic PCR assay targeting type A minicircles (Lai et al. 2008; Carnes et al. 2015). Therefore, a VSG JN 2118Hu primer set was used in the JN 2118Hu PCR assay for identifying non-RoTat 1.2 *T. b. evansi*, which amplified a 273 bp DNA segment within *T. b. evansi* JN 2118Hu coding region that lacked similarity with any other known trypanosome sequence (Ngaira et al. 2005). Notably, 42/91 (46.2%) samples were positive in VSG JN 2118Hu PCR. However, Ngaira et al. (2005) suggested that the VSG JN 2118Hu, which was initially identified in a *T. b. evansi* strain from Kenya, is a distinctive marker for non-RoTat 1.2 *T. b. evansi,* Birhanu et al. (2016) reported that the JN 2118Hu VSG PCR was less specific since *T. b. brucei* and *T. b. gambiense* yielded positive amplification in this PCR. Hence, we further verified the absence of maxicircle DNA in JN 2118Hu VSG PCR-positive samples with the ND5 PCR (Dean et al. 2013). We have included the detection of a single copy control gene (GPI-PLC PCR) to corroborate that adequate parasite genomic material would have been present within the reaction to give a positive result. This means that the amplification of GPI-PLC demonstrates *T. b. evansi* when enough parasitic material is present within the sample to effectively amplify the VSG gene if present. A negative PCR result may signify the absence of VSG gene or simply mean that insufficient genomic material is present (Picozzi et al. 2008). Both RoTat 1.2 VSG PCR and JN 2118Hu VSG PCR failed to detect two samples that showed positive amplification by GPI-PLC PCR although Ngaira et al. (2005) recorded 100% sensitivity of the JN 2118Hu VSG PCR. The JN 2118Hu VSG PCR succeeded to detect non-RoTat 1.2 *T. b. evansi* for the first time in Egyptian camels. Previous studies reported that RoTat VSG PCR could not detect *T. b. evansi* in some infected animals from different species (Elhaig and Sallam 2018; Behour et al. 2019). This could support presence of non-RoTat 1.2 *T. b. evansi*t in Egypt since it has been detected in some African countries, including Sudan, Ethiopia, Chad, and Kenya (Ngaira et al. 2005; Hagos et al. 2009; Salim et al. 2011; Birhanu et al. 2015; Sánchez et al. 2015). Notably, all tested samples were negative with EVAB PCR that indicates absence of *T. b. evansi* type B minicircles. An earlier study in Egypt reported that *T. b. evansi* prevalence in camels usingTBR and RoTat 1.2 PCR were 54.5% and 21.8%, respectively (Elhaig and Sallam 2018). A lower prevalence of *Trypanosoma* spp in camels was reported in Oman, which was 78/95, 77%; 30/95, 31.6%; 8/95, and 8.4% using TBR, ITS, and RoTat 1.2 PCRs, respectively, with no amplification obtained by EVAB PCR** (**Al-Kharusi et al. 2022). Moreover, 39.5% and 36.6% molecular prevalence were obtained using ITS-1 PCR in Saudi Arabia and Sudan, respectively (Salim et al. 2011; Metwally et al. 2021). In Algeria, 11.2% molecular prevalence was reported with RoTat 1.2 PCR (Boushaki et al. 2019).

The outcomes of the different PCR assays performed for each sample (Table 3) revealed that only 47 out of 80 positive samples could be classified as *T. b. evansi*. These samples were successfully amplified using TBR, ITS-1, and RoTat 1.2 or JN 2118Hu VSG PCR. The remaining 33 samples could be classified as Trypanozoon. Nineteen out of the 33 samples were only TBR PCR positive which may be due to the high sensitivity of the TBR primers. Five samples were TBR and ITS-1 PCR positive and neither gave positive amplification by RoTat1.2 PCR nor JN 2118Hu PCR. Also, 9 samples were only TBR and JN 2118Hu PCR positive. This might be due to the variations in the sensitivity of the PCR primers. Notably, all RoTat 1.2 PCR-positive samples were also ITS-1 PCR-positive. The nine samples that tested positive with JN 2118Hu VSG PCR were ITS-1 negative. This indicates the consistency between ITS-1 and RoTat 1.2 PCR assays for detecting *T. b. evansi*. Regarding typing of *T. b. evansi* in Egypt (Table 4), RoTat 1.2 *T. b. evansi* is more prevalent than non-RoTat 1.2 *T. b. evansi* in El Nubariyah and Giza districts. However, only non-RoTat 1.2 *T. b. evansi* was detected in Aswan district, which might indicate that the origin of the new non-RoTat 1.2 *T. b. evansi*, is the quarantined animals coming from Sudan. *T. b. evansi* type A was most prevalent in Ethiopia, with 11.7% molecular prevalence. However, only 0.53% of it was *T. b. evansi* type B (Birhanu et al. 2015). Recent studies in Oman and Algeria did not find any evidence for *T. b. evansi* type B (Boushaki et al. 2019; Al-Kharusi et al. 2022).

The phylogenetic tree (Fig. 7) indicated that the *T. b. evansi* sequence isolates targeting RoTat 1.2 VSG were closely related, representing that this genetic sequence is highly conserved among these isolates. Our isolate is significantly related to the Indian and Indonesian isolates and is a unique sequence isolate of this region from camels. The alignment of VSG JN 2118Hu PCR sequences showed that these isolates were identical and indicated sequence repetition. Although no significant similarity was found between the obtained sequence and that was published by Ngaira et al. (2005), our sequence showed 3 nucleotide deletion compared with the sequence size of Ngaira et al. (2005). The restriction fragments yielded by *Hae* III enzyme (using NEBcutter program) exposed that one of the restriction fragments (149 bp) has the same size with that obtained by Ngaira et al. (2005) yet different sequence. Absence of similarity of VSG JN 2118Hu PCR sequence with non-Rotat1.2 sequence obtained by Ngaira et al. (2005) and sequence data on GenBank hypothesizes detection of new structure of non RoTat1.2 *T. b. evansi.* This is expected, since *T. b. evansi* is considered as petite mutant of *T. brucei* (Lai et al. 2008). It is clear now that type A *T. b. evansi* isolates but RoTat1.2 negative are more common than anticipated, which could lead to a high frequency of false-negative results by current techniques depend on RoTat1.2 VSG. Therefore, combination of RoTat1.2 VSG based PCR and alternatives like VSG JN 2118Hu PCR and A281del PCR could detect the majority of *T. b. evansi* (Urakawa et al, 2001; Ngaira et al. 2004, 2005; Kamidi et al. 2017). Noteworthy, Ngaira et al. (2005) raised a question about considering the non-RoTat1.2 *T. b. evansi* belongs to type B group according to the speculation of Claes et al. (2004). In conclusion, the TBR PCR assay is the most sensitive among the four PCR assays used to detect *T. b. evansi* in camels. Regarding the typing of *T. b. evansi*, RoTat 1.2* T. b. evansi* is more prevalent in Egypt, while a new non-RoTat 1.2 *T. b. evansi* was detected for the first time. *T. b. evansi* RoTat 1.2 ARRI isolate is significantly closer to the Indian and Indonesian isolates and is considered a unique isolate from the camels. However, we could not find any similarity between the VSG JN 2118Hu PCR sequences with the GenBank sequences, which is a limitation of this study. The new genetic structure could be a variant of of non-RoTat 1.2 *T. b. evansi* so further research on lager sample size is required to verify the new sequences to corroborate the prevalence of non-RoTat1.2 *T. b. evansi* in Egypt.

## Data Availability

All data generated or analyzed during this study are included in this published article [and its supplementary information files].
